# Supplementary material for: Decoding numeracy and literacy in the human brain: insights from MEG and MVPA
Source: Sci Rep. 2023 Jul 6;13:10979. doi: 10.1038/s41598-023-37113-0 (PMC10326015; doi:10.1038/s41598-023-37113-0)
Supplement: Supplementary file 1 — Supplementary Figure 1. [file 41598_2023_37113_MOESM1_ESM.docx]

**Supplementary Section:**

**
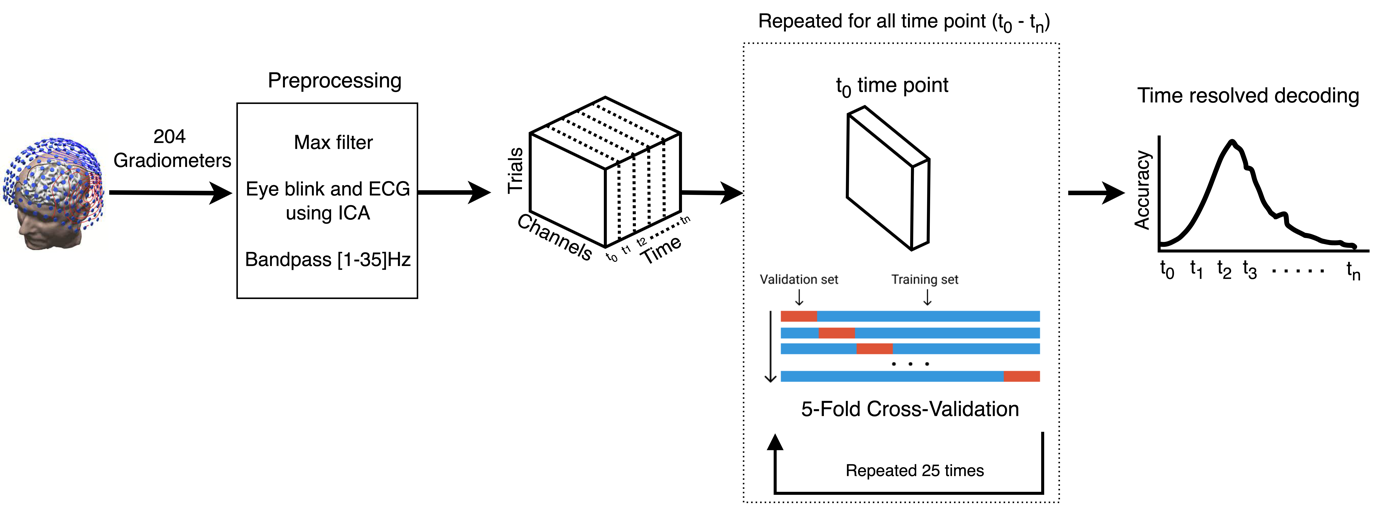
**

*Supplementary Figure 1*: Graphical representation of the data analysis pipeline used for time-resolved decoding.
